# Protodioscin mitigates cyclophosphamide-induced renal toxicity in HEK-293 cells by regulating endoplasmic reticulum stress, antioxidant defense, and apoptotic pathways

**DOI:** 10.1371/journal.pone.0357227

**Published:** 2026-09-21

**Authors:** Ahmad Reza Ahmadi, Touraj Zamir Nasta, Ardeshir Abbasi, Mohammad Reza Tabandeh, Cyrus Jalili

**Affiliations:** 1 Medical Biology Research Center, Health Technology Institute, Kermanshah University of Medical Sciences, Kermanshah, Iran; 2 Medicinal Plant Research Center, Ahvaz Jundishapur University of Medical Sciences, Ahvaz, Iran; 3 Department of Immunology, Factually of Medical Sciences, Tarbiat Modares University, Tehran, Iran; 4 Department of Biochemistry and Molecular Biology, Faculty of Veterinary Medicine, Shahid Chamran University of Ahvaz, Ahvaz, Iran; 5 Stem Cells and Transgenic Technology Research Center, Shahid Chamran University of Ahvaz, Ahvaz, Iran; Weill Cornell Medicine, UNITED STATES OF AMERICA

## Abstract

Cyclophosphamide (CTX) is a widely used chemotherapeutic agent with well-established efficacy; however, its clinical use is limited by dose-dependent adverse effects, particularly nephrotoxicity. Protodioscin (Prot), a bioactive saponin derived from plants of the Dioscoreaceae family, has demonstrated notable antioxidant and anti-inflammatory properties. This study aimed to evaluate the protective effects of Prot against CTX-induced cytotoxicity in HEK-293 cells. Primary HEK-293 kidney cells were exposed to the IC50 concentration of CTX in the presence or absence of Prot (5 and 10 µM) for 24 hours. Cell viability was assessed using the MTT assay. Gene expression of Bax and Bcl-2 was measured by real-time PCR assay, while protein expression of Bax, cleaved caspase-3, Bcl-2, ATF6, PERK, GRP78, CHOP, p-eIF2α, p-PERK, and cleaved ATF6 were evaluated by Western blot analysis. Oxidative stress parameters, including CAT, SOD, GPx, GSH, MDA, and NO, were quantified using spectrophotometric assays, and kidney injury markers (KIM-1 and cystatin C) were measured by ELISA. Prot decreased Bax and caspase 3 expression and increased Bcl-2 expression in HEK-293 cells exposed to CTX, which was accompanied by reduced apoptosis. Treatment with 5 and 10 µM Prot significantly reduced the expression of ATF6, PERK, GRP78, CHOP, p-eIF2α, p-PERK and Cleaved- ATF6 proteins in CTX-exposed HEK-293 cells. Additionally, treatment with Prot significantly increased catalase, GPx, GSH, and SOD levels, while reducing KIM-1, cystatin C, MDA, and NO levels in HEK-293 cells subjected to CTX. These findings suggest that Prot exerts a protective role against CTX-induced renal injury in HEK-293 cells by modulating apoptotic pathways, attenuating endoplasmic reticulum stress, and improving antioxidant indices.

## 1. Introduction

Cyclophosphamide (CTX) is a chemotherapeutic and immunosuppressive agent widely used in the treatment of various malignancies, as an immune suppressor before bone marrow transplantation [1] or as a chemotherapy agent against various cancers such as breast cancer, lung carcinoma, multiple myeloma, and leukemia [2] as well as autoimmune disorders [3]. As an alkylating agent, CTX exerts its cytotoxic effects primarily through cross-linking DNA, thus disrupting cellular replication and transcription processes [4].

Despite its therapeutic efficacy, the clinical use of cyclophosphamide is often complicated by dose-dependent toxicities that affect various organ systems, especially the kidneys [5]. The kidneys play a vital role in the metabolism and excretion of CTX and its active metabolites, and renal cells are highly sensitive to CTX-induced damage [6]. This toxicity manifests through a complex interplay of cellular mechanisms such as oxidative stress, inflammation, and endoplasmic reticulum (ER) stress, leading to cellular dysfunction and apoptosis in the kidney parenchyma [7]. Understanding the complex molecular pathways underlying CTX-induced nephrotoxicity is critical for developing effective strategies to reduce these adverse effects and improve patient outcomes [8].

The physiology of CTX-induced nephrotoxic injury is multifactorial and not fully elucidated, but the predominant mechanism involves oxidative stress-mediated damage [9]. During CTX metabolism, reactive oxygen species (ROS) are produced, including superoxide anions, hydroxyl radicals, and hydrogen peroxide, which overwhelm the endogenous antioxidant defense systems. This imbalance leads to lipid peroxidation, protein oxidation, DNA damage, and mitochondrial injury in kidney cells [10]. Studies have shown decreased activity of antioxidant enzymes such as superoxide dismutase (SOD), catalase, and glutathione peroxidase (GPX) in kidney tissues following CTX exposure, indicating reduced antioxidant capacity [11]. Additionally, CTX metabolites like acrolein contribute to direct tubular toxicity and inflammation, exacerbating kidney damage [12].

Apoptosis, or programmed cell death, is a crucial end-point consequence of oxidative damage and plays a central role in CTX-induced cytotoxicity in the kidney [13]. The intrinsic apoptosis pathway is precisely regulated by the BCL-2 family of proteins, where pro-apoptotic members like BAX increase the permeability of the mitochondrial outer membrane, while anti-apoptotic members such as BCL-2 exert cellular protective effects [14]. An imbalance favoring BAX over BCL-2 leads to the release of cytochrome c and activation of caspases, ultimately resulting in apoptosis [15]. Increased expression of BAX, caspase and decreased BCL-2 in HEK-293 cells under oxidative stress and CTX treatment has been documented, indicating a disrupted regulation of apoptosis in nephrotoxicity [16].

Recent evidence shows that endoplasmic reticulum (ER) stress is also a mechanistic factor in CTX-induced kidney injury [17]. The ER plays a central role in protein folding and calcium homeostasis; disturbances in its function activate the unfolded protein response (UPR) to restore cellular homeostasis or, if the stress remains unresolved, induce apoptosis [18]. Key UPR components include activating transcription factor 6 (ATF6), Protein kinase R-like ER kinase (PERK), C/EBP homologous protein (CHOP), and glucose-regulated protein 78 (GRP78). In this context, activation of PERK through phosphorylation (p-PERK) leads to phosphorylation of eIF2α (p-eIF2α), resulting in translational attenuation and reduced protein load within the ER [19]. Similarly, ATF6 is activated via proteolytic cleavage to generate its active form (ATF6s), which translocates to the nucleus to regulate ER stress-responsive genes [20]. Overactivation of UPR elements, particularly CHOP induction and sustained PERK/eIF2α signaling, facilitates apoptotic pathways and contributes to cellular dysfunction [21,22]. Examining ER stress markers in HEK-293 cells exposed to CTX is vital for clarifying the role of this pathway in cytotoxicity [23].

Due to the pivotal role of oxidative stress and apoptosis in CTX-induced nephrotoxicity, antioxidant and cytoprotective agents have attracted researchers’ attention to mitigate kidney damage [24]. Phytochemicals with rich biological properties have emerged as promising candidates [25]. Among these, Prot, a steroidal saponin derived from medicinal plants such as Tribulus terrestris, stands out. This compound has gained significant scientific interest due to its broad spectrum of pharmacological properties, including anticancer, anti-inflammatory, and immunomodulatory activities [26]. Emerging research shows that Prot has high antioxidant potential, capable of scavenging free radicals and elevating endogenous antioxidant enzymes. Its molecular structure, which includes a steroidal aglycone and a sugar chain, facilitates diverse biological interactions [27]. Preclinical studies have demonstrated that Prot exerts its protective effects via multiple mechanisms, including modulation of cellular signaling pathways involved in cell proliferation, apoptosis, and inflammation [28]. Antioxidant, anti-inflammatory, and anti-apoptotic activities have been demonstrated in various cellular and animal models [29]. Prot regulates mitochondrial function, increases endogenous antioxidant enzyme activity, and modulates apoptosis gene expression, identifying it as a potential renal protective agent [30]. Considering the proven roles of oxidative stress, ER stress, and apoptosis in CTX-induced nephrotoxicity, the diverse pharmacological profile of Prot makes it a promising candidate for investigation as a renal protective agent.

Therefore, this study aims to systematically evaluate the therapeutic efficacy of Prot against cyclophosphamide-induced cytotoxicity in the HEK293 renal cell line, a well-known laboratory model for studying renal cell biology and toxicology. By exposing cultured HEK293 cells to CTX followed by treatment with various concentrations of Prot, The study evaluates cell viability and apoptosis using the TUNEL assay and the expression of BAX, BCL-2, and cleaved caspase-3. ER stress markers (ATF6, cleaved ATF6, PERK, p-PERK, GRP78, CHOP, and p-eIF2α) are analyzed by Western blot. Oxidative and antioxidant status is assessed by measuring catalase, malondialdehyde (MDA), nitric oxide (NO), superoxide dismutase (SOD), glutathione peroxidase (GPx), and reduced glutathione (GSH), and kidney injury is evaluated by measuring kidney injury molecule-1 (KIM-1) and cystatin C. This approach aims to elucidate the antioxidant, anti-apoptotic, and ER stress-modulating mechanisms of Prot against CTX-induced nephrotoxicity in HEK-293 Cells.

## 2. Materials and methods

### 2.1. Ethics statement

This work was supported by the Vice-Chancellor for Research of Kermanshah University of Medical Sciences, Kermanshah, Iran (Grant number: 4030872).

### 2.2. Materials and reagents

DMEM/F12 culture medium (Gibco, USA) was supplemented with fetal bovine serum (FBS; Gibco, USA), penicillin (100 U/mL), streptomycin (100 μg/mL), and 2 mM L-glutamine (Gibco, USA). Trypan blue (Sigma-Aldrich, USA) was used for cell staining. MTT reagent and dimethyl sulfoxide (DMSO) were purchased from Sigma-Aldrich (USA). Cyclophosphamide (Sigma-Aldrich, USA) and Protodioscin (CAS No. 55056-80-9; Sigma-Aldrich, USA) were prepared according to previously standardized protocols. RNA extraction and cDNA synthesis kits (Parstous Co, Tehran, Iran) were used according to the manufacturer’s instructions. Primers were designed based on sequences available in the NCBI database and validated using Primer3 software. Protein concentration was quantified using the Bradford Protein Assay Kit (Bio-Rad, USA). The catalase, MDA, SOD GSH, and NO assay kits (Kiazist, Iran), as well as the GPx assay kit (Randox Laboratories Ltd., Crumlin, UK), were used for biochemical analyses. ELISA kits for KIM-1(SL1031Hu; SUNLONG Biotechnology, China) and cystatin C (SL0575Hu; SUNLONG Biotechnology, China) were used to determine the corresponding biomarker levels.

### 2.3. Cell culture

Human embryonic kidney cell line HEK-293 was obtained from the Cell Bank of Pasteur Institute of Iran (ATCC CRL-1573). Cells were stained with trypan blue, and the number of viable cells was determined. The culture medium DMEM/F12 supplemented with 10% FBS, penicillin/streptomycin, and 2 mM glutamine was used for cell culture at 37°C in a humidified incubator (Padideh Nogen, Iran) with 5% CO2. The incubator temperature was maintained at 37°C throughout the experiment, and the medium was refreshed every two days prior to experiments.

### 2.4. Cell viability by MTT assay

Cells were treated with CTX at concentrations of 1, 10, 25, 50, 100, and 150 µM for 24 hours to assess viability. HEK-293 cells (5 × 10^4^) were seeded in 96-well plates containing 200 µl DMEM/F12 medium with 10% FBS and incubated at 37°C in 5% CO_2_. After 24 hours, 10 µl of MTT solution (Sigma-Aldrich, MO, USA) was added to each well and incubated at 37°C for 4 hours in the dark. The supernatant was removed and 100 µl of DMSO added to each well. After complete solubilization of the crystals in DMSO, the absorbance of each well was measured at 540 nm using an ELISA reader (BioTek, USA). The following formula was used to calculate cell viability:


$$\begin{array}{c} (\text{Abs test cells }/\text{ Abs control cells})\;\times \text{ 100 }=\;(\%)\text{ cell viability} \end{array}$$

### 2.5. Experimental groups

Following cell viability assessment and determination of the IC50 concentration of CTX, experimental groups were established for subsequent analyses. The experimental groups included untreated control cells, cells treated with Prot alone at 10 µM as the negative control, cells treated with CTX alone at 54 µM as the positive control, cells co-treated with 5 µM Prot and 54 µM CTX, and cells co-treated with 10 µM Prot and 54 µM CTX. The selected concentrations of Prot were based on previous studies and showed no cytotoxic effects on the cells [31,32]. For the TUNEL and apoptosis assays, an additional H₂O₂-treated group was included as a positive apoptosis control. Based on previously published studies, cells in this group were exposed to 1 mM H₂O₂ for 4 h before apoptosis assessment.

### 2.6. qRT-qPCR analysis of apoptosis-related gene expression

The treated cells were detached using 0.25% Trypsin (Bioidea, Tehran, Iran) and then rinsed with sterile PBS three times. The cells were centrifuged at 1000 × g for 5 min. The cells were precipitated, and the supernatant was removed. Total RNA was extracted from HEK-293 Cells using a Total RNA Extraction Kit (Parstous Co, Tehran, Iran) as recommended by the manufacturer. The purity and concentration of RNA were assessed by measuring the 260/280 OD ratio using an Eppendorf Cuvette G1.0 micro volume measuring cell (Eppendorf, Hamburg, Germany). cDNA was synthesized from 1 μg of total RNA using the Easy™ cDNA Synthesis Kit (Parstous Co, Tehran, Iran) according to the producer’s instructions. Real-time PCR was performed using the Lava96 Real-time PCR Detection System (DaAnGene Co Ltd, Guangzhou, China) by the 2X SYBR Green Real Time PCR (Parstous, Tehran, Iran). The relative expression level of the target genes was normalized to the GAPDH gene. All qRT-PCR reactions were performed in triplicates. No-template controls (NTC) and no-reverse-transcription control were included in the qPCR assay. Relative quantification was performed according to the comparative 2^-ΔΔCt^ method, using Lava96 software 3.2. The primer sequences used in this study are presented in Table 1.

**Table 1:** Sequence of primers that used in the current study.

Gene name	Sequences	Size (bp)	GenBank accession No
GAPDH	F: CAGCCTCAAGATCATCAGCAATG R: CATGAGTCCTTCCACGATACCA	100	XM_002046.7
*BAX*	F: AAGAAGCTGAGCGAGTGTCT R: TGCCGTCAGAAAACATGTCAG	142	NM_001291428.2
*Bcl-2*	F: AGAGTTGCTTTACGTGGCCT R: AGCACCACTGCATTTCAGGA	116	NM_000633.3

### 2.7. Assessment of protein expression by Western blot analysis

Protein expression of Bax, Bcl-2, Caspase 3, ATF6, PERK, GRP78, CHOP, p-PERK, p-eIF2α, and Cleaved- ATF6 in HEK-293 Cells exposed to CTX and Prot was measured by Western blot analysis [33]. Following experimental treatment, the cell pellets lysed in 100 µl of lysis buffer (Tris-HCl 50 mM, NaCl 150 mM, Triton X-100 0.1%, NaF 1 mM) supplied with protease inhibitor cocktails (Sigma-Aldrich, MO, USA) for 30 min on ice. The protein concentration of the cell lysate was quantified using the Bradford protein assay (Bio-Rad, USA). Proteins were denatured, and a total of 10 μg of protein was loaded onto a 10% SDS-PAGE gel for electrophoresis. The proteins were then transferred onto polyvinylidene fluoride (PVDF) membranes (Amersham, USA) and blocked overnight at 4°C with 5% BSA (Sigma, USA) in Tris-buffered saline (TBS, Sigma, USA) containing 0.05% Tween-20 (Sigma, USA). Subsequently, the membranes were incubated overnight with primary antibodies including: anti-Bax (Sc-7480,Santa Cruz Biotechnology, USA), anti-Bcl-2(Sc-7382,Santa Cruz Biotechnology, USA), anti-Caspase 3(#9662, Cell Signaling Technology, USA), anti-ATF6 (#65880, Cell Signaling Technology, USA), anti-PERK (Sc377400,Santa Cruz Biotechnology, USA), anti-GRP78 (Sc13539,Santa Cruz Biotechnology, USA), anti-CHOP (#2895, Cell Signaling Technology, USA), anti-p-PERK (Sc-32577,Santa Cruz Biotechnology, USA), anti-p-eIF2α (#3398, Cell Signaling Technology, USA), anti-cleaved ATF6 (853101, BioLegend San Diego,CA, USA) and GAPDH (#5174, Cell Signaling Technology, USA) diluted 1:500 in blocking solution. The membranes were incubated with an appropriate secondary antibody conjugated to horseradish peroxidase diluted 1:1000 in block solution for 2 h (NB500–420, Novus Biologicals, CO, USA). After three additional washing steps, protein signals were visualized using an ECL detection kit (Parstous, Iran). Protein loading was normalized against GAPDH immunoreactivity (5174, Cell Signaling Technology, USA). The blots were detected using a Fusion X chemiluminescence imaging system (Vilber, USA), and optical density was quantified using Fusion X software. Data were expressed as fold change relative to the control group.

### 2.8. Assessment of Apoptosis by the TUNEL Assay

Following 24 h of treatment with CTX and Prot, cells were washed twice with PBS and fixed with 4% formaldehyde for 30 min at room temperature. After fixation, the cells were washed with PBS and permeabilized according to the manufacturer’s instructions. Apoptosis was assessed using a TUNEL assay kit (E-CK-A334, Elabscience Biotechnology, USA). The samples were incubated with the TUNEL reaction mixture for 45 min at 37°C in a humidified chamber protected from light. Cells treated with H₂O₂ were used as the positive control, while samples incubated without the TUNEL reaction reagent served as the negative control. After incubation, the cells were washed to remove excess reagent and counterstained with an appropriate nuclear dye. TUNEL-positive cells were visualized using a fluorescence microscope, and images were captured from randomly selected fields in each experimental group. The apoptotic index was calculated as the percentage of TUNEL-positive nuclei relative to the total number of nuclei.

### 2.9. Measurement of antioxidant/oxidant factors

The levels of GSH, MDA, and NO, as well as the activities of antioxidant enzymes including Catalase, GPx, and SOD, were measured in HEK-293 cells exposed to CTX and Prot using spectrophotometric assays. Following 24 h of treatment, HEK-293 cells were detached using 0.25% trypsin-EDTA and centrifuged at 1000 × g for 5 min. The resulting cell pellets were washed with cold PBS and lysed in PBS supplemented with a protease inhibitor cocktail. After incubation on ice, the lysates were centrifuged at 12,000 × g for 15 min at 4°C, and the supernatants were collected for further biochemical analyses.Total protein concentration was determined using the Bradford protein assay method (Bio-Rad, USA), according to the manufacturer’s instructions. The levels of MDA, NO, CAT, and SOD were measured using commercial assay kits (Kiazist, Iran), whereas GPx activity was measured using a commercial assay kit (Randox Laboratories Ltd., Crumlin, UK), according to the manufacturers’ instructions.

GSH levels were measured according to Hu et al. (1994) based on the reaction of thiol groups with 5,5′-dithiobis (2-nitrobenzoic acid) (DTNB) to form the yellow-colored 5-thio-2-nitrobenzoic acid (TNB), which is proportional to GSH concentration [34]. Briefly, samples, standards, and blank were incubated with phosphate buffer (pH 7.6) and DTNB at room temperature for 5 min, and absorbance was read at 412 nm. GSH levels were calculated using a standard curve and expressed as μmol/mg protein

Results for GSH, MDA, and NO were normalized to total protein content and expressed as nmol/mg protein, while Catalase, GPx, and SOD activities were expressed as U/mg protein.

### 2.10. Measurement of KIM-1 and Cystatin C

The protein levels of KIM-1 and Cystatin C in renal cells exposed to cyclophosphamide and prooxidant were measured by ELISA kits (Sunlong Biotechnology, Inc) as recommended by the manufacturer. All samples were run in one assay to avoid inter-assay variation. The analysis was performed in triplicate. The intra assay coefficients of variation of both hormones were < 10%.

### 2.11. Statistical analysis

Statistical analyses were performed using GraphPad Prism version 10. Data normality was assessed using the Shapiro–Wilk test. Normally distributed data were analyzed by one-way analysis of variance (ANOVA) and are presented as the Mean ± standard deviation (SD). When significant differences were detected by ANOVA, Tukey’s multiple comparison test was applied to compare the mean values among experimental groups. Statistical significance was defined as follows: *p < 0.05, **p < 0.01, ***p < 0.001, and ****p < 0.0001. All experiments were performed in triplicate.

## 3. Results

### 3.1. Effect of CTX on the viability of HEK-293 cells

**Fig 1:**
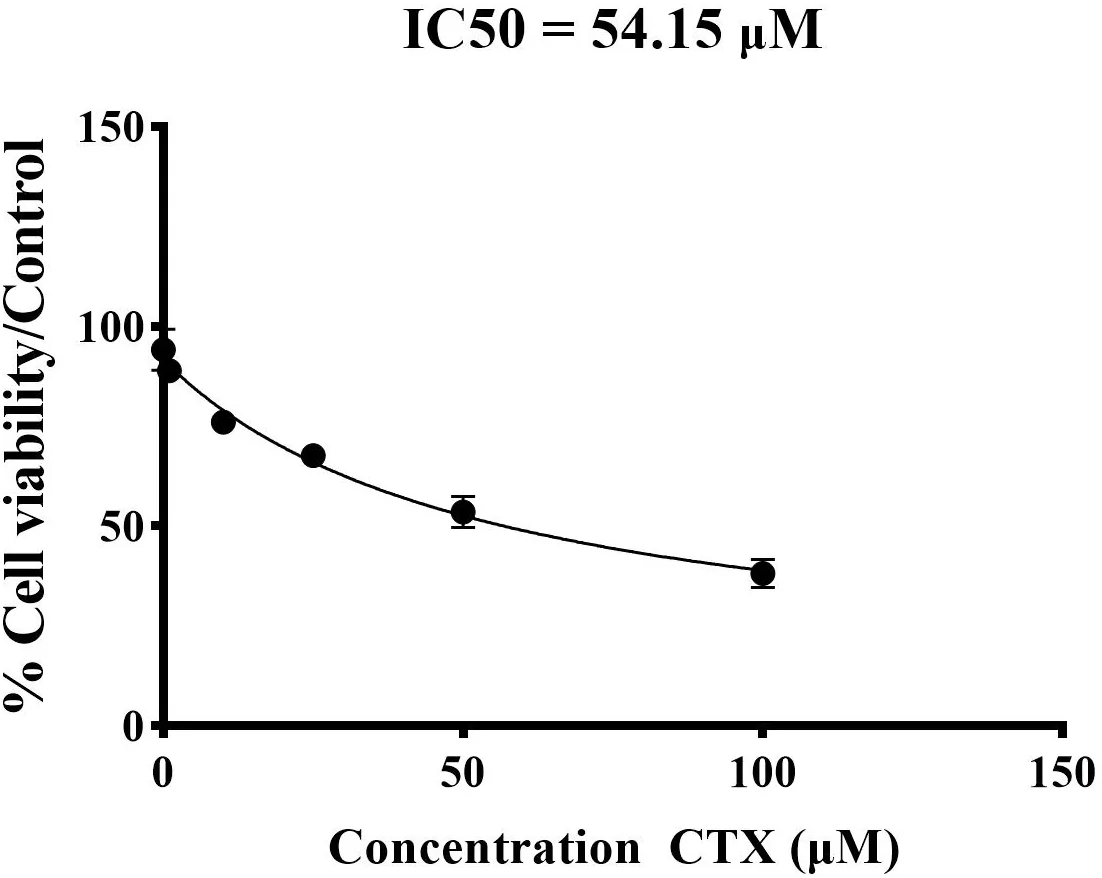
Cellular viability (MTT assay) of HEK-293 cells exposed to various concentrations of cyclophosphamide (CTX) for 24 hours.

The results shown in Fig 1 indicated that CTX significantly reduced cell viability in the HEK-293 cell line in a dose-dependent manner compared to the control group. The calculated IC₅₀ value was 54.15 µM, indicating a strong cytotoxic effect of CTX on HEK-293 cells.(Fig 1).

### 3.2. The effect of Prot on the expression of apoptotic genes and proteins in HEK-293 cells exposed to CTX

**Fig 2:**
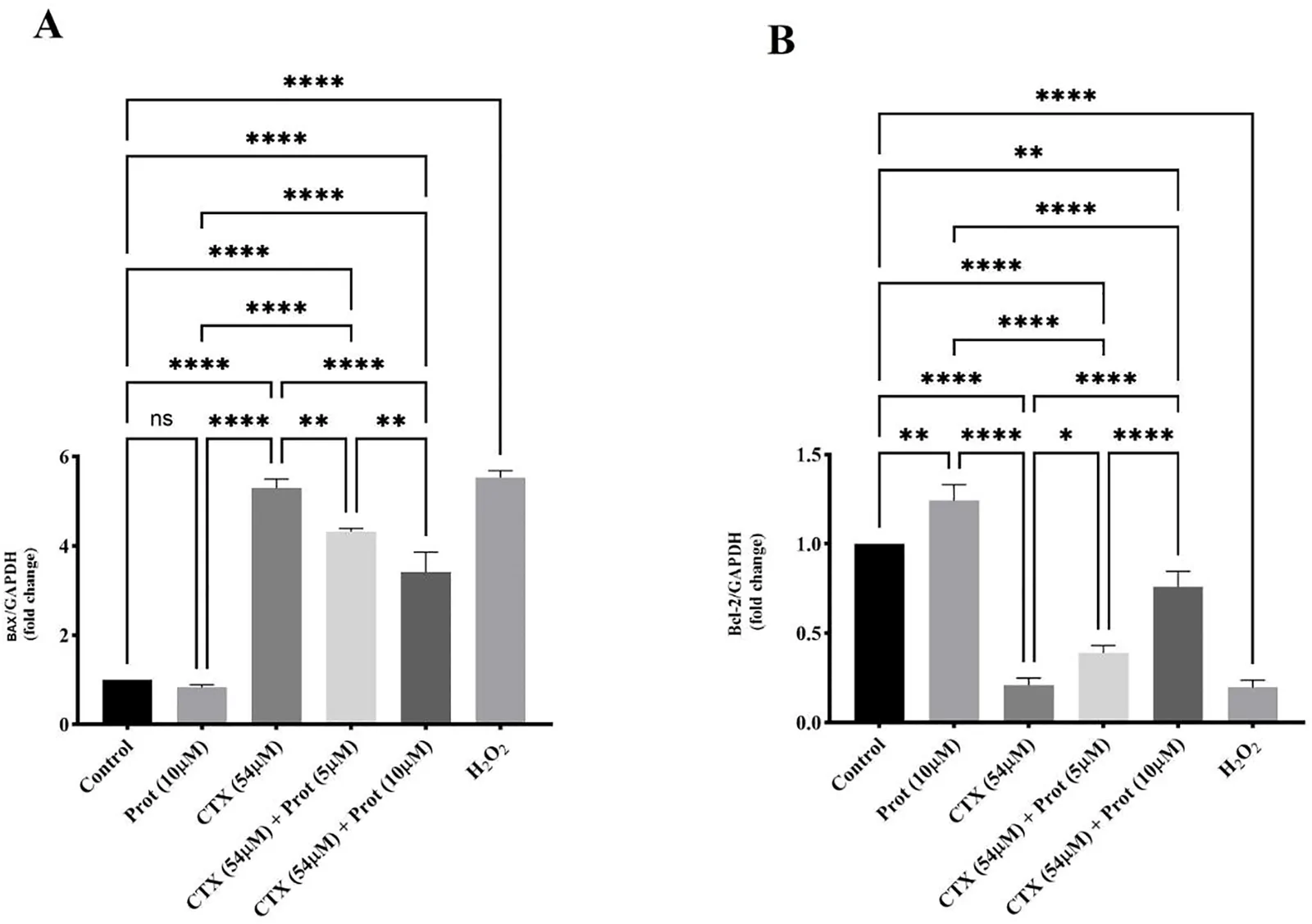
Effects of Prot on the expression of apoptosis-related genes in CTX-exposed HEK-293 cells. Quantitative analysis of BAX (A), and BCL-2 (B). Control (untreated group), Prot (10 µM Protodioscin), CTX (54 µM Cyclophosphamide), CTX + Prot 5 (54 µM Cyclophosphamide + 5 µM Protodioscin), CTX + Prot 10 (54 µM Cyclophosphamide + 10 µM Protodioscin), H2O2 (positive control for Apoptosis). GAPDH was used as a housekeeping gene. Results are presented as Mean ± SD of three independent experiments. *, **, ***, and **** denote statistical significance between groups at p < 0.05, p < 0.01, p < 0.001, and p < 0.0001 respectively.

*BAX* expression were significantly elevated in the CTX group compared with the control group (P < 0.0001), while *Bcl-2* expression was significantly decreased (P < 0.0001). HEK-293 cells treated with Prot at concentrations of 5 and 10 μM significantly reduced *BAX* expression (P < 0.0001, P < 0.01), while significantly increasing *Bcl-2* expression relative to the CTX group (P < 0.0001, P < 0.05,). *BAX* expression was significantly reduced in the CTX + Prot (10 μM) group compared with the CTX + Prot (5 μM) group (P < 0.01), whereas *Bcl-2* expression was significantly increased (P < 0.0001). H₂O₂ exposure significantly increased the expression levels of *BAX*, while markedly decreasing *Bcl-2* expression compared with the control group (P < 0.0001). This confirmed that apoptotic pathways are activated under oxidative stress conditions (Fig 2 A- B)

**Fig 3:**
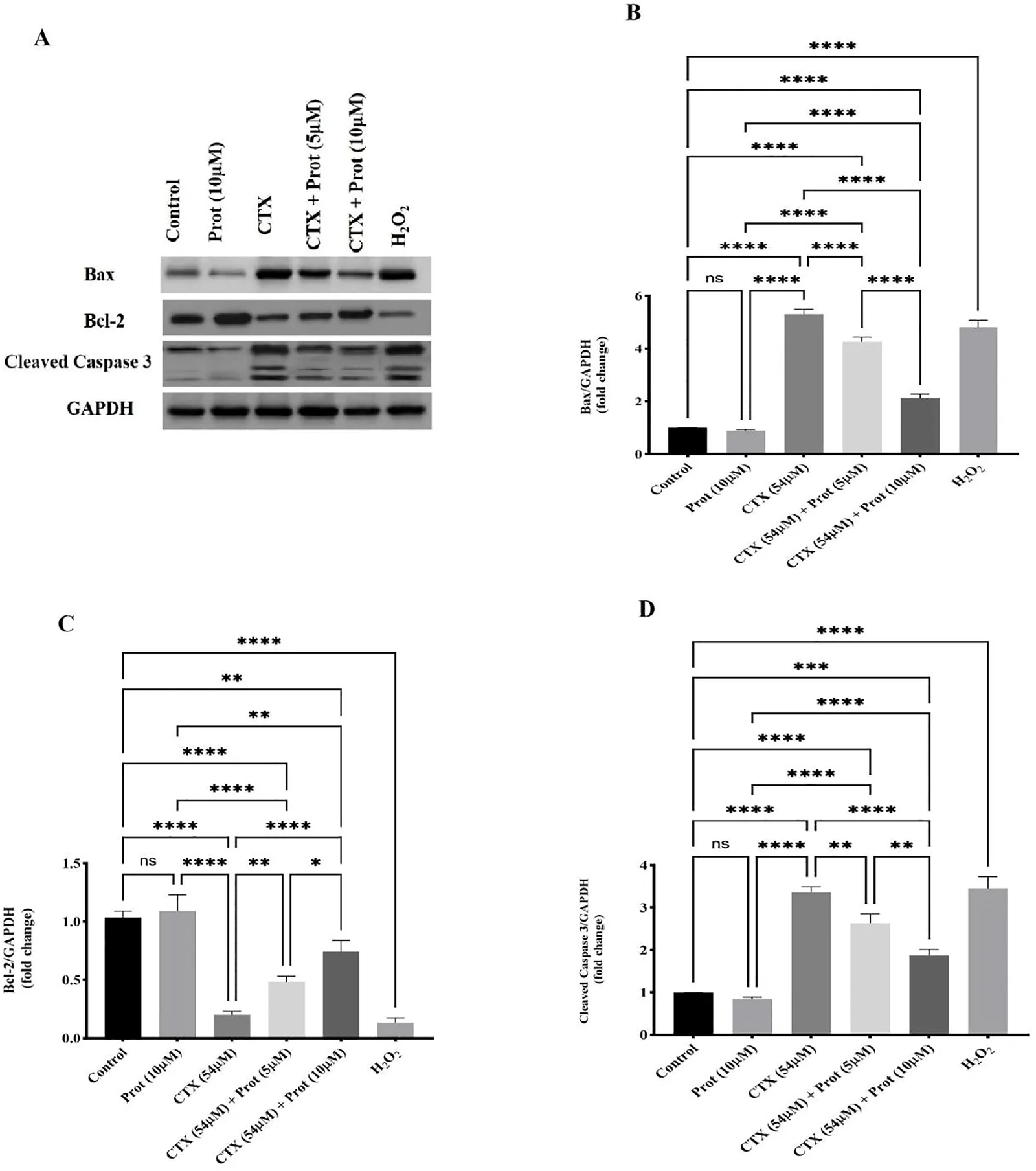
Effects of Prot on the expression of apoptosis-related proteins in HEK-293 cells exposed to CTX. (A)Western blot analysis. (B-D)Quantitative analysis of BAX, BCL-2 and Cleaved Caspase-3 proteins expression. Control (untreated group), Prot (10 µM Protodioscin), CTX (54 µM Cyclophosphamide), CTX + Prot 5 (54 µM Cyclophosphamide + 5 µM Protodioscin), CTX + Prot 10 (54 µM Cyclophosphamide + 10 µM Protodioscin), H2O2 (positive control for Apoptosis). Protein levels were assessed by Western blot analysis. GAPDH was used as a calibrator protein. Results are presented as Mean ± SD of independent experiments. *, **, ***, and **** denote statistical significance between groups at p < 0.05, p < 0.01, p < 0.001, and p < 0.0001 respectively.

Western blot analysis showed that CTX significantly increased the protein expression levels of Bax and cleaved Caspase-3 compared to the control group (P < 0.0001). In contrast, Bcl-2 expression was markedly reduced in the CTX group (P < 0.0001). Treatment with Prot (5 and 10 µM) significantly decreased the protein expression levels of Bax and cleaved Caspase-3(P < 0.01 to P < 0.0001), while increasing Bcl-2 expression compared to the CTX group (P < 0.01, P < 0.0001). Bax and cleaved caspase-3 protein expression were significantly reduced (P < 0.0001, P < 0. 01), whereas Bcl-2 protein expression was significantly increased (P < 0.05) in the CTX + Prot (10 μM) group compared with the CTX + Prot (5 μM) group. H₂O₂ treatment elevated the protein levels of Bax and cleaved Caspase-3, whereas Bcl-2 protein expression was reduced relative to the control group (P < 0.0001). (Fig 3 A- D)

### 3.3. Effect of Prot on CTX-Induced Apoptosis in HEK-293 Cells Assessed by TUNEL Assay

To evaluate CTX-induced apoptosis and the protective effect of Prot, TUNEL staining combined with DAPI counterstaining was performed.

Qualitative analysis (Panel A) showed minimal TUNEL-positive signals in the control and Prot-only (10 µM) groups. In contrast, CTX treatment markedly increased the number of TUNEL-positive cells, indicating strong induction of apoptosis. Co-treatment with Prot significantly reduced TUNEL-positive signals compared to the CTX group, with a more pronounced effect at the 10 µM dose. Overall, these findings indicate that Prot attenuates CTX-induced apoptosis in a dose-dependent manner.H₂O₂ (as positice control) exposure resulted in a marked increase in TUNEL-positive cells compared to the control group.

**Fig 4:**
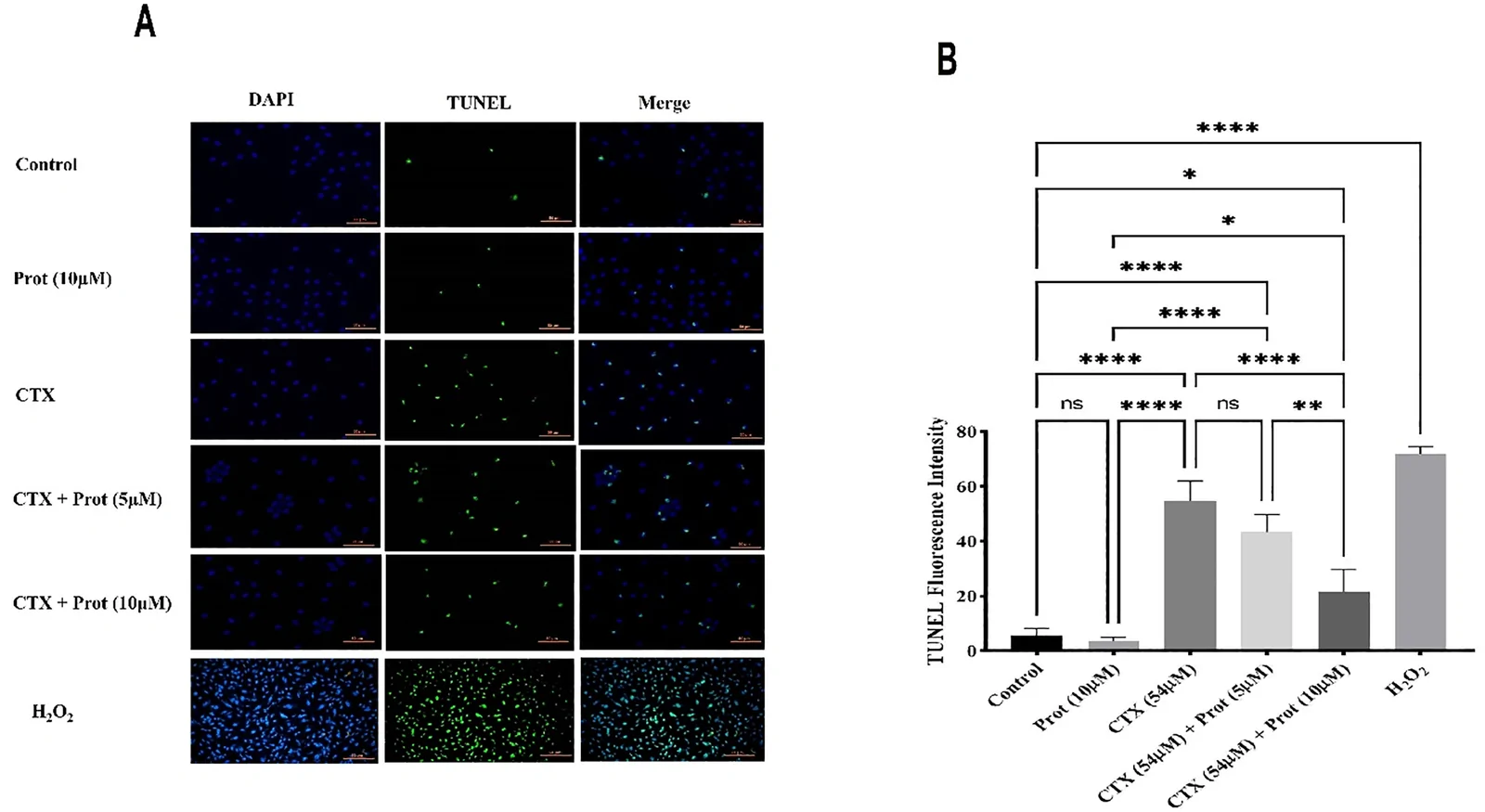
Effects of Prot on apoptosis in CTX-exposed HEK-293 cells assessed by the TUNEL assay. (A) Representative fluorescence images of DAPI staining (blue), TUNEL-positive cells (green), and merged images. (B) Quantitative analysis of TUNEL fluorescence intensity. Experimental groups included: Control (untreated), Prot (10 µM Protodioscin), CTX (54 µM Cyclophosphamide), CTX + Prot 5 (54 µM Cyclophosphamide + 5 µM Protodioscin), CTX + Prot 10 (54 µM Cyclophosphamide + 10 µM Protodioscin), and H₂O₂ (positive control for apoptosis). Results are presented as mean ± SD of independent experiments. *, **, ***, and **** indicate statistical significance at P < 0.05, P < 0.01, P < 0.001, and P < 0.0001, respectively.

Quantitative analysis (Panel B) statistically confirmed these observations. The TUNEL fluorescence intensity in the CTX group showed a highly significant increase compared to the control group (P < 0.0001). Co-treatment of cells with 5 and 10 µM Prot reduced fluorescence intensity compared to the CTX group (P < 0.0001). TUNEL fluorescence intensity was significantly reduced in the CTX + Prot (10 μM) group compared with the CTX + Prot (5 μM) group (P < 0.01).The protective effect of Prot was dose-dependent, with the reduction of apoptosis being stronger at 10 µM than at 5 µM. (Fig 4 A-B).

### 3.4. The effect of Prot the expression of ERS- related proteins in HEK-293 cells exposed to CTX

**Fig 5:**
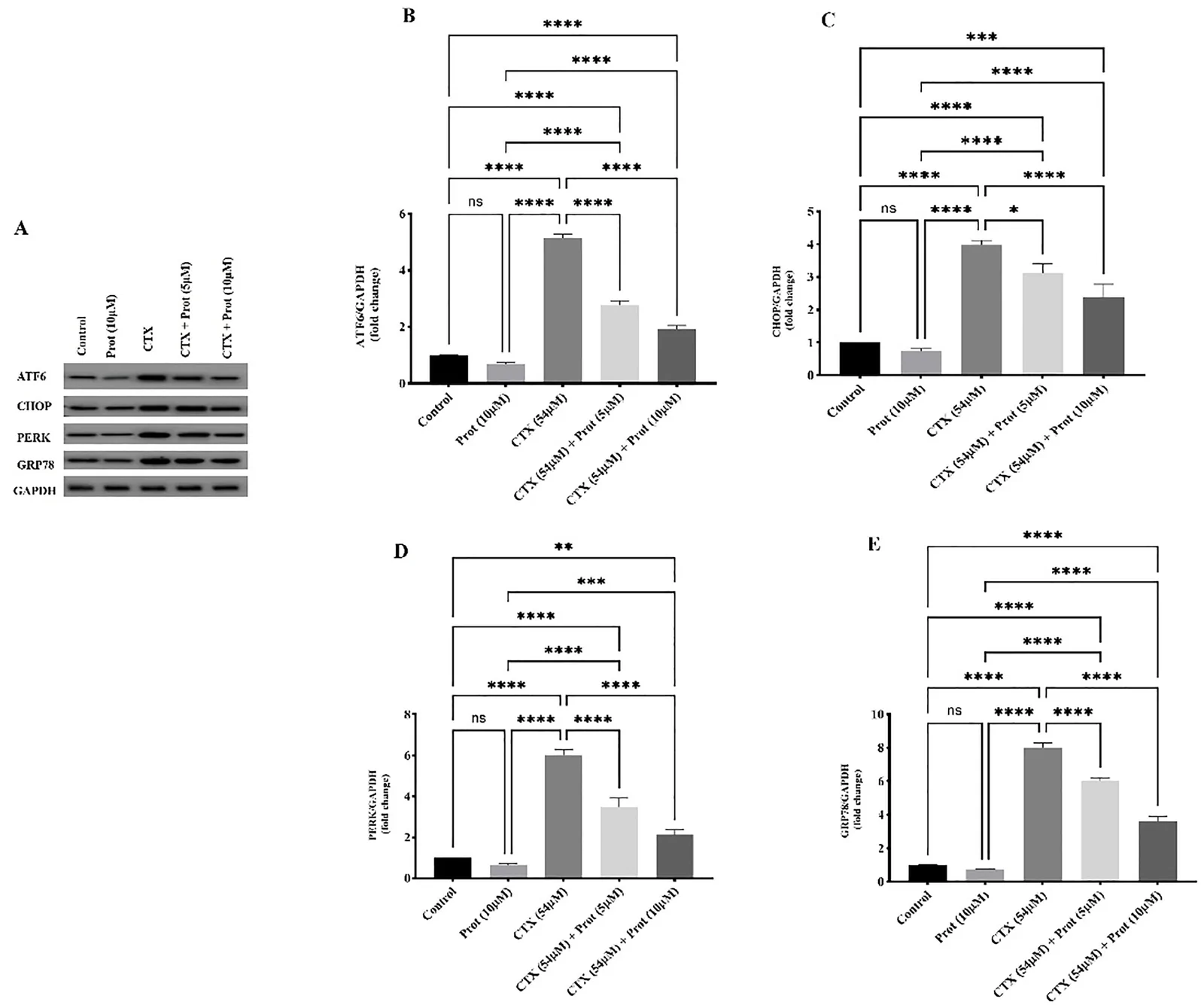
Effects of Prot on the expression of endoplasmic reticulum stress–related proteins in HEK-293 cells exposed to CTX. (A)Western blot analysis. (B-E) Quantitative analysis of ATF6, CHOP, PERK, and GRP78. Control (untreated group), Prot (10 µM Protodioscin), CTX (54 µM Cyclophosphamide), CTX + Prot 5 (54 µM Cyclophosphamide + 5 µM Protodioscin), CTX + Prot 10 (54 µM Cyclophosphamide + 10 µM Protodioscin). Protein levels related to ER stress were assessed by Western blot analysis. GAPDH was used as a calibrator protein. Results are presented as Mean ± SD of independent experiments. *, **, ***, and **** denote statistical significance between groups at p < 0.05, p < 0.01, p < 0.001, and p < 0.0001 respectively.

Western blot analysis was employed to evaluate the effects of Prot on the protein expression of key endoplasmic reticulum (ER) stress markers. The results confirmed that CTX significantly increased the protein levels of PERK and ATF6, GRP78, and CHOP, compared with the control group (P < 0.0001). Treatment of HEK-293 cells with Prot (5 and 10 µM) significantly downregulated PERK, ATF6, GRP78, and CHOP protein expression compared with the CTX group (P < 0.0001). Protein levels of PERK, ATF6, GRP78, and CHOP were significantly reduced in the CTX + Prot (10 μM) group compared with the CTX + Prot (5 μM) group (P < 0.001, P < 0.0001, P < 0.0001, and P < 0.05, respectively). (Fig 5 A–E).

The results demonstrated that CTX significantly increased the protein expression levels of cleaved ATF6, p-PERK, and p-eIF2α compared with the control group (P < 0.0001).

**Fig 6:**
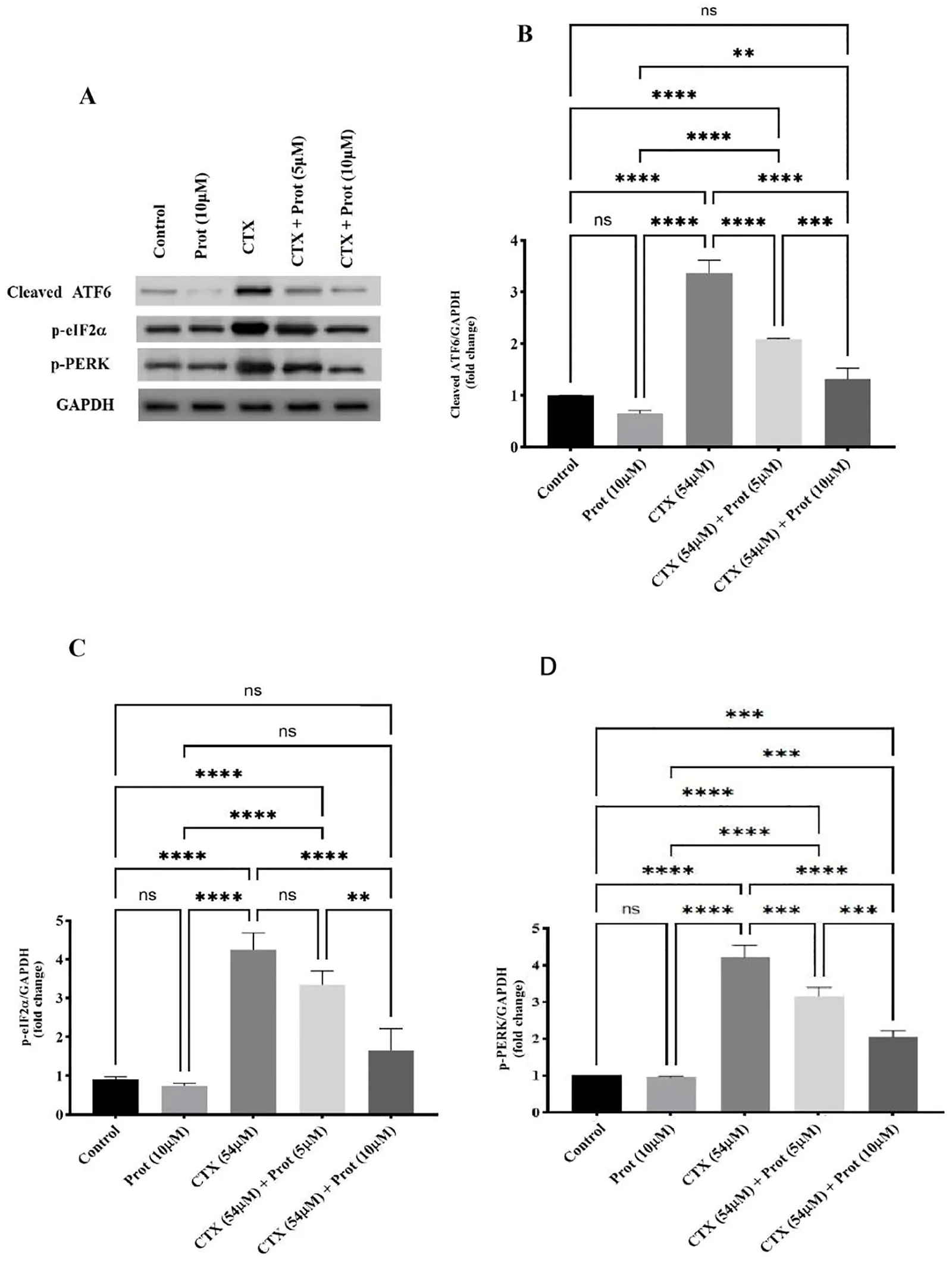
Effects of Prot on the expression of phosphorylated ER stress–related proteins in HEK-293 cells exposed to CTX. (A)Western blot analysis. (B-D) Quantitative analysis of cleaved ATF6, p-eIF2α, and p-PERK,. Control (untreated group), Prot (10 µM Protodioscin), CTX (54 µM Cyclophosphamide), CTX + Prot 5 (54 µM Cyclophosphamide + 5 µM Protodioscin), CTX + Prot 10 (54 µM Cyclophosphamide + 10 µM Protodioscin).Protein levels related to ER stress were assessed by Western blot analysis. GAPDH was used as a calibrator protein. Results are presented as Mean ± SD of independent experiments. *, **, ***, and **** denote statistical significance between groups at p < 0.05, p < 0.01, p < 0.001, and p < 0.0001 respectively.

Treatment with Prot (5 and 10 µM) significantly reduced the protein expression levels of cleaved ATF6 and p-PERK compared with the CTX group (P < 0.001,P < 0.0001). In contrast, for p-eIF2α, a significant reduction was observed only at 10 µM, while no significant difference was detected at 5 µM compared with the CTX group. Protein levels of cleaved ATF6, p-PERK, and p-eIF2α were significantly reduced in the CTX + Prot (10 μM) group compared with the CTX + Prot (5 μM) group (P < 0.001, P < 0.001, and P < 0.01, respectively). (Fig 6 A–D).

### 3.5. The effect of Prot on antioxidant/oxidative markers in HEK-293 cells exposed to CTX

A more detailed investigation of CTX toxicity mechanisms and the protective effects of protodiosin was performed by assessing key oxidative stress markers.The results showed that CTX administration induced severe oxidative stress in HEK-293 cells, accompanied by disruption of the cellular antioxidant defense system and a notable increase in lipid peroxidation.

**Fig 7:**
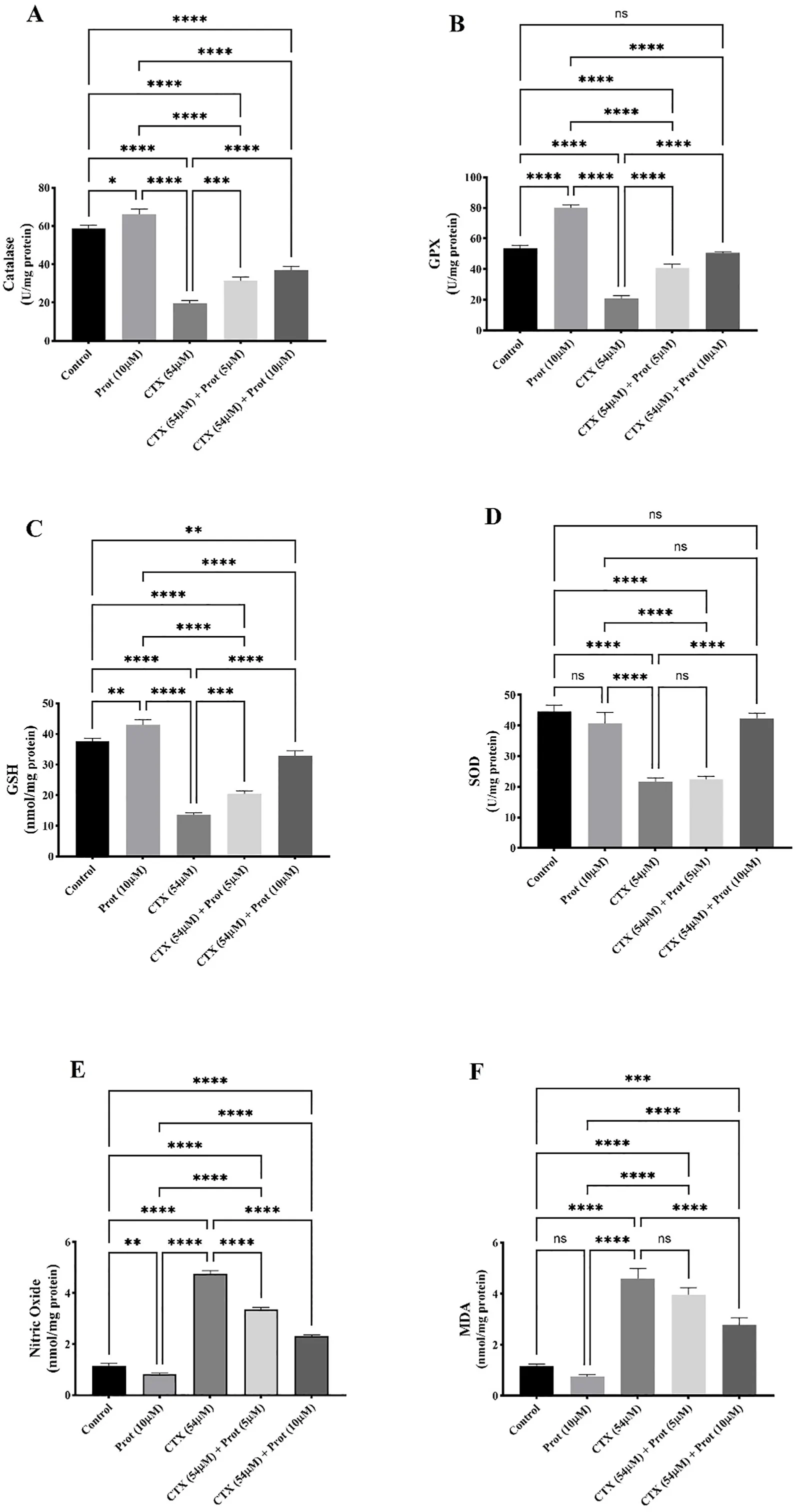
Effects of Prot on Catalase (A), GPx (B), GSH (C), SOD (D), NO (E) and MDA (F) levels in HEK-293 cells exposed to CTX. Control (untreated group), Prot (10 µM Protodioscin), CTX (54 µM Cyclophosphamide), CTX + Prot 5 (54 µM Cyclophosphamide + 5 µM Protodioscin), CTX + Prot 10 (54 µM Cyclophosphamide + 10 µM Protodioscin). Results are presented as Mean ± SD of three independent experiments. *, **, ***, and **** indicate statistical significance between groups at p < 0.05, p < 0.01, p < 0.001, and p < 0.0001, respectively.

The activities of Catalase and GPx were significantly decreased in the CTX group compared with the control group (P < 0.0001). Treatment with Prot at both 5 and 10 µM significantly increased Catalase and GPx activities compared with the CTX group (P < 0.0001) (Fig 7A–B). Similarly, SOD activity was significantly reduced in the CTX group compared with the control group (P < 0.0001). Prot at 10 µM significantly increased SOD activity compared with the CTX group, whereas Prot at 5 µM did not produce a significant change (Fig 7 D).

GSH levels were significantly decreased in the CTX group compared with the control group (P < 0.0001). Prot treatment at both 5 and 10 µM significantly increased GSH levels compared with the CTX group (P < 0.0001) (Fig 7 C). The activities of GPx and SOD and the GSH level were significantly increased in the CTX + Prot (10 μM) group compared with the CTX + Prot (5 μM) group (P < 0.001, P < 0.0001, and P < 0.0001, respectively). (Fig 7 A- D).

In contrast, MDA and NO levels were significantly elevated in the CTX group compared with the control group (P < 0.0001). Treatment with Prot at 5 and 10 µM significantly reduced MDA and NO levels compared with the CTX group (P < 0.0001). MDA and NO levels were significantly reduced in the CTX + Prot (10 μM) group compared with the CTX + Prot (5 μM) group (P < 0.0001 and P < 0.01, respectively). (Fig 7 E–F).

### 3.6. The effect of Prot on kidney specific injury markers in HEK-293 cells exposed to CTX

**Fig 8:**
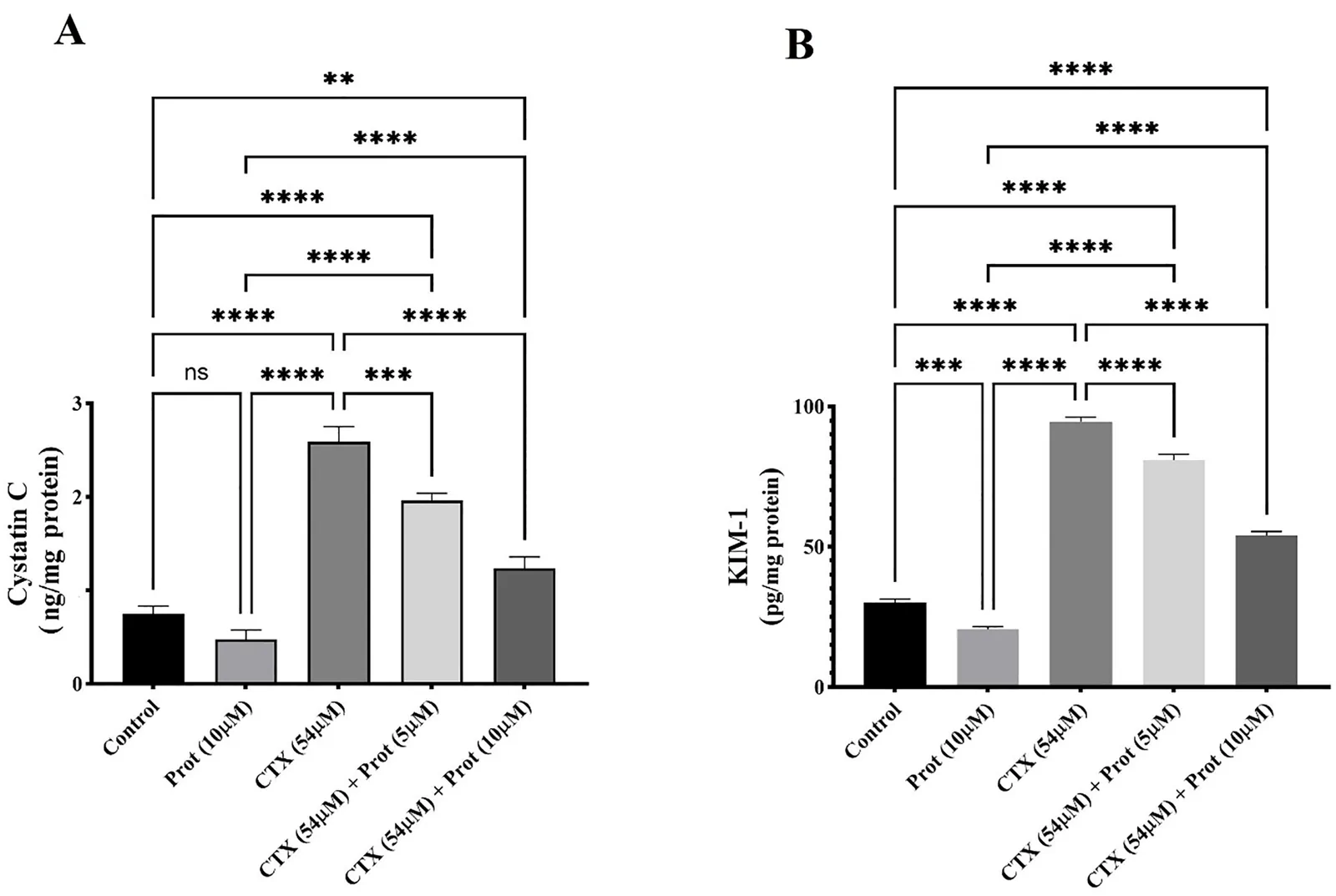
Effects of Prot t on Cystatin C (A) and KIM-1 (B) levels in HEK-293 cells exposed to CTX measured by ELISA. Control (untreated group), Prot (10 µM Protodioscin), CTX (54 µM Cyclophosphamide), CTX + Prot 5 (54 µM Cyclophosphamide + 5 µM Protodioscin), CTX + Prot 10 (54 µM Cyclophosphamide + 10 µM Protodioscin). Results are presented as Mean ± SD of three independent experiments. *, **, ***, and **** indicate statistical significance between groups at p < 0.05, p < 0.01, p < 0.001, and p < 0.0001, respectively.

KIM-1 and cystatin C levels were significantly increased in the CTX group compared with the control group (P < 0.0001). Treatment with Prot at both 5 and 10 µM significantly reduced KIM-1 and cystatin C levels compared with the CTX group (P < 0.0001). KIM-1 and cystatin C levels were significantly reduced in the CTX + Prot (10 μM) group compared with the CTX + Prot (5 μM) group (P < 0.0001 and P < 0.001, respectively). (Fig 8A-B).

## 4. Discussion

CTX, as an important chemotherapeutic and immunosuppressive drug, has widespread applications in the treatment of various cancers and autoimmune diseases [35,36]. Its therapeutic efficacy is attributed to its ability to cross-link DNA strands, thereby inhibiting DNA replication and transcription, ultimately leading to cell cycle arrest and apoptosis in rapidly dividing cells [37]. However, a significant limitation in its clinical use is dose-dependent toxicity, which affects various off-target tissues, with nephrotoxicity being a concerning adverse effect [38]. The kidneys play a vital role in the metabolism and excretion of CTX and its active metabolites, and renal cells are highly susceptible to CTX-induced damage [39]. This toxicity manifests through a complex interplay of cellular mechanisms, including oxidative stress, inflammation, and ER stress, resulting in cellular dysfunction and apoptosis in the renal parenchyma [40]. Understanding the intricate molecular pathways underlying CTX-induced nephrotoxicity is crucial for developing effective strategies to mitigate these detrimental effects and improve patient outcomes. Therefore, the identification and development of compounds capable of reducing this toxicity are of great importance. Prot is a natural steroidal glycoside with antioxidant, anti-apoptotic, and ER stress-inhibitory properties and may be considered a therapeutic candidate to alleviate CTX-induced adverse effects [27]. Accordingly, this study aimed to systematically evaluate the therapeutic efficacy of Prot against cyclophosphamide-induced cytotoxicity in the HEK293 renal cell line, a well-established in vitro model for studying renal cell biology and toxicology [41].

Our study initially demonstrated that CTX caused a significant dose-dependent reduction in HEK293 cell viability, with an IC50 value of 54.15 µM. This finding aligns with numerous previous reports indicating the mortality and toxicity of cyclophosphamide in renal cells and other cellular models [42]. The known mechanism of CTX involves the generation of active DNA-alkylating metabolites and cellular induction of DNA damage, ultimately leading to cell death [43]. Similar findings in various studies have shown that CTX induces oxidative damage and apoptosis in cells [44,45].

The increased expression of BAX and decreased expression of BCL-2 in the CTX-treated group indicate activation of the intrinsic apoptotic pathway [46]. BAX is a pro-apoptotic protein, whereas Bcl-2 is anti-apoptotic, and the ratio between them typically determines cell fate. It has been established that a high BAX/Bcl-2 ratio stimulates apoptosis [47]. Treatment with Prot at concentrations of 5 and 10 µM reversed these changes by decreasing BAX expression and increasing Bcl-2. These findings were further confirmed by Western blot analysis, which showed increased BAX and cleaved Caspase-3 protein expression and decreased BCL-2 expression in the CTX group. Prot (5 and 10 µM) reversed these changes at both the mRNA and protein levels, indicating that its protective effect is partly mediated through inhibition of apoptosis.This suggests that Prot exerts part of its protective effect by regulating these key apoptotic regulators, thereby inhibiting CTX-induced apoptosis. The anti-apoptotic effect observed aligns with other studies that have demonstrated that natural compounds can restore the balance of Bcl-2 family proteins to protect against cellular damage [48,49]. For instance, icariin (ICA), a component of Epimedium, has been shown to prevent apoptosis by regulating the expression of caspase-3, Bcl-2, and BAX in CTX-induced cellular injury models in HT22 and HEK293 cells [50]. In another study, natural olive‑derived compounds were shown to protect HEK293 cells by reducing oxidative stress and modulating apoptotic pathways, including the downregulation of caspase‑3 and the restoration of the Bcl‑2/BAX balance [51].

The TUNEL assay further corroborated the gene expression findings related to apoptosis, revealing a significant increase in TUNEL-positive cells in the CTX group, indicative of extensive DNA fragmentation and widespread apoptosis. Treatment with Prot significantly reduced the number of TUNEL-positive cells in a dose-dependent manner, with the 10 µM dose exhibiting a more pronounced protective effect. These direct evidences of reduced apoptosis suggest that Prot interferes with the cell death process induced by CTX. The reduction of apoptotic cells following Prot treatment is critical, as apoptosis is a well-recognized mechanism underlying CTX-induced organ toxicity. Similar anti-apoptotic effects of Prot have been reported in other studies [52].

ER stress plays a key role in CTX-induced cellular damage ATF6s [53]. Administration of CTX significantly increased the protein levels of PERK, ATF6, GRP78, CHOP, p-PERK, p-eIF2α, and indicating a robust ER stress response. These proteins serve as markers of activated ER stress pathways and the initiation of ER stress-mediated apoptosis [54]. They are also key indicators of the unfolded protein response (UPR) pathway, which is activated in response to ER stress [55]. Dereli̇ et al. (2026) reported that cyclophosphamide induces substantial ER stress in renal tissue, as reflected by elevated levels of GRP78, PERK, IRE1, eIF2α, ATF4, and CHOP. PERK and ATF6 are membrane sensors that initiate downstream signaling cascades [56], Activation of PERK via phosphorylation is an early ER stress response that phosphorylates eIF2α, reducing global protein synthesis and limiting misfolded protein accumulation in ER stress [19]. However, sustained PERK/eIF2α activation promotes pro-apoptotic signaling and cellular dysfunction. In parallel, ATF6 is activated by proteolytic cleavage to form cleaved-ATF6, which translocates to the nucleus and regulates ER stress–responsive genes [57]. GRP78 (BiP) is an ER chaperone whose upregulation indicates ER stress, while CHOP is a pro-apoptotic transcription factor induced during prolonged stress [58]. In this study, increased p-PERK, p-eIF2α, and cleaved-ATF6 after CTX exposure confirm activation of the PERK and ATF6 branches of the unfolded protein response.

The study by Ben Salem et al. demonstrated that zearalenone induces strong ER stress in HCT116 and HEK293 cells via activation of UPR markers (GRP78, GADD34, ATF4, XBP1, and CHOP), leading to apoptotic cell death. Importantly, the study highlighted the protective role of natural compounds (crocin and quercetin) in attenuating ER stress by suppressing GRP78/GADD34 expression and downstream UPR signaling, thereby reducing ER stress–mediated apoptosis. In our study, Prot treatment attenuated CTX-induced ER stress in HEK293 cells, as evidenced by the downregulation of key UPR and ER stress-related proteins, including ATF6, CHOP, GRP78, p-PERK, p-eIF2α, and cleaved ATF6. These findings suggest a cytoprotective effect of Prot through modulation of ER stress–associated signaling pathways. Prot-mediated regulation of ER stress is crucial, as ER stress is a key contributor to kidney disease and drug-induced nephrotoxicity. For instance, cyclosporine A (CsA) induces ER stress and activates UPR-associated apoptotic pathways in HEK293 and proximal tubular cells. [59]. Genetic suppression of the UPR in HEK293 cells reduces CsA-induced apoptotic responses, underscoring the significance of ER stress in nephrotoxicity.

An important mechanism of CTX-induced nephrotoxicity is excessive ROS generation and subsequent oxidative stress [60]. In this study, CTX exposure in HEK293 cells significantly reduced antioxidant defenses, including Catalase, GPX, and reduced GSH. Treatment with CTX at both doses effectively restored catalase, GPX, and GSH levels, indicating enhancement of the endogenous antioxidant system. SOD activity was also reduced by CTX, but was significantly increased only at 10 µM Prot, suggesting a dose-dependent effect.In addition, Prot markedly decreased CTX-induced MDA and NO elevation, reflecting reduced lipid peroxidation and oxidative damage. These results are consistent with previous reports showing that compounds such as amifostine, coenzyme Q10, carvacrol, and formononetin can enhance antioxidant enzyme activity in CTX-induced nephrotoxicity models [61]. Previous studies have also highlighted the antioxidant potential of Prot in protecting against oxidative stress [62]. Overall, these findings demonstrate that Prot effectively attenuates CTX-induced oxidative stress in HEK293 cells by enhancing antioxidant defenses and reducing oxidative damage markers.

To evaluate the extent of kidney damage and the protective potential of Prot, we measured the levels of KIM-1 and cystatin C. Both KIM-1 and cystatin C are sensitive and reliable biomarkers of kidney injury; KIM-1 is a marker of tubular damage, whereas cystatin C is an accurate indicator of glomerular function [63,64]. The significant elevation of both markers in the CTX group indicated substantial cellular damage and impaired renal function at the cellular level. Prot treatment markedly prevented the increase of these markers, significantly lowering cystatin C and KIM-1 levels compared to the CTX-only group. This strong evidence suggests that Prot can attenuate CTX-induced damage in renal cells. The reduction of these specific kidney injury markers by Prot reinforces its nephroprotective effects and indicates its potential to preserve the integrity and function of both tubular and glomerular compartments. The observed decrease in kidney injury markers with Prot aligns with findings from other studies where various protective agents improved renal function and reduced markers such as creatinine and urea in CTX-induced nephrotoxicity models [65].

Therefore, the observed protective effects of Prot are likely multifactorial, involving direct regulation of apoptotic pathways, reduction of endoplasmic reticulum stress, and robust enhancement of the cellular antioxidant defense system.

### Limitations, strengths, and future perspectives

The present study was performed in an in vitro model using only HEK-293 cells; therefore, the findings do not fully reflect the complexity of renal tissue and physiological responses in vivo. Validation of these findings in additional renal cell lines and animal models remains necessary before clinical translation. Despite these limitations, the study provides a comprehensive evaluation of cyclophosphamide-induced renal injury through the simultaneous assessment of oxidative stress, apoptosis, endoplasmic reticulum stress, and kidney injury biomarkers. The integration of molecular, biochemical, and functional analyses strengthens the mechanistic interpretation of the protective effects of Prot. Future research should focus on in vivo validation and further exploration of the therapeutic potential of protodioscin as a nephroprotective agent during cyclophosphamide treatment

## 5. Conclusion

**Fig 9:**
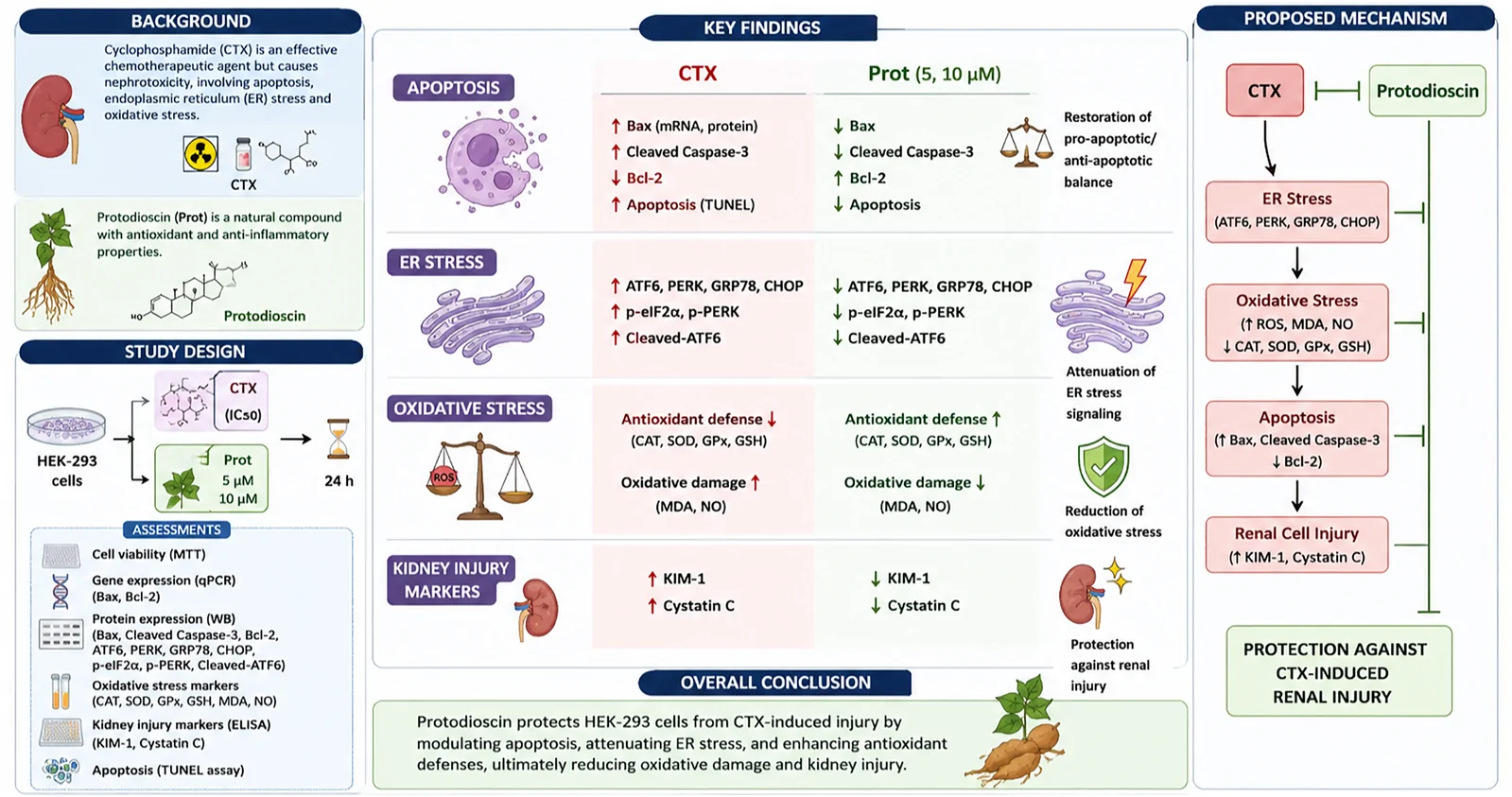
Graphical abstract illustrating the protective effects of protodioscin against cyclophosphamide-induced renal injury in HEK-293 cells through modulation of apoptosis, endoplasmic reticulum stress, and oxidative stress pathways.

Based on the present findings, protodioscin (Prot) demonstrates significant protective effects against cyclophosphamide (CTX)-induced toxicity in HEK-293 cells. Prot attenuates renal cell injury by regulating apoptotic pathways, suppressing ER stress, and enhancing the cellular antioxidant defense system. The underlying mechanisms of these effects are summarized in the graphical abstract (Fig 9). Overall, these results indicate the potential of Prot as an adjunctive therapeutic agent for reducing CTX-associated nephrotoxicity in chemotherapy and support further preclinical and clinical evaluation.
