# Supplementary material for: Protodioscin mitigates cyclophosphamide-induced renal toxicity in HEK-293 cells by regulating endoplasmic reticulum stress, antioxidant defense, and apoptotic pathways
Source: PLoS One. 2026 Sep 21;21(9):e0357227. doi: 10.1371/journal.pone.0357227 (PMC13592597; doi:10.1371/journal.pone.0357227)
Supplement: Supplementary file 1 [file pone.0357227.s002.docx]

July, 05, 2026

Dear Editor-in-Chief of PLOS One

My co-authors and I sincerely appreciate the reviewers’ encouraging, critical, and constructive comments on our manuscript entitled “Protodioscin Mitigates Cyclophosphamide-Induced Renal Toxicity in HEK-293 Cells by regulating Antioxidant and Apoptotic Pathways”. In response to their recommendations, and considering the limitations of time and available experimental facilities, we performed additional Western blot analyses for the apoptosis-related proteins BAX, BCL-2, and cleaved caspase-3, as well as the ER stress signaling proteins cleaved ATF6, phosphorylated PERK (p-PERK), and phosphorylated eIF2α (p-eIF2α). These new data have been incorporated into the revised manuscript. In addition, H₂O₂ was included as a positive control in the apoptosis and TUNEL assays, and a graphical abstract has been added to the revised manuscript. We are grateful for the insightful feedback and valuable suggestions that have helped us improve the quality and clarity of our paper.

Major final revisions have been made in accordance with the reviewer’s comments==============================

**Response to editor’s**

Q1. The study was conducted using a single cell line, which significantly undermines the generalizability of the conclusions. Given that the manuscript contains exclusively in vitro data, the authors should explicitly discuss the limitations of the study.

R1.We thank the reviewer for this important comment. We agree that the use of a single cell line limits the generalizability of the findings. This limitation has now been explicitly acknowledged and discussed in the limitations section of the revised Discussion

Q2. The Methods section requires additional detail, including the amount of total protein loaded, antibody dilutions, primer sequences, and related experimental parameters. Alternatively, appropriate references to previously published methods should be provided.

R2. Thank you for this valuable comment. The Methods section has been revised and expanded to include the requested experimental details

Q3. The Results section is very concise and should be expanded to provide more descriptive interpretation and clearer conclusions from the presented data.

R3. Thank you for this suggestion. The Results section has been revised and expanded to provide a more comprehensive description and interpretation of the findings.

Q4. Figure 1 – Is the X-axis (CTX concentration) presented on a logarithmic scale?

R4. Thank you for your valuable comment. The X-axis in Figure 1 is presented on a linear scale, as indicated by the equal spacing between 0, 50, 100, and 150 μM and the inclusion of zero, which is not compatible with a logarithmic scale. A linear scale was chosen to display the raw data across the tested concentration range directly

Q5. Most experiments lack appropriate positive controls (e.g., apoptosis, oxidative stress, or ER-stress inducers) as well as corresponding inhibitors/negative controls. Their inclusion would strengthen the interpretation of the results.

R5. Thank you for this valuable suggestion. In response, a positive control group treated with H₂O₂ was included in the apoptosis-related experiments. The expression of apoptosis-associated genes and proteins, as well as TUNEL assay results, were evaluated in this positive control group and compared with the experimental groups. The corresponding data have been added to the Results section of the revised manuscript to strengthen the interpretation of the findings.

Q6. Assessment of Bax and Bcl-2 mRNA levels alone is insufficient; protein expression levels should also be evaluated. In addition, it is unclear why Bax and Bcl-2 measurements are presented in Figure 2 when apoptosis itself is demonstrated only later in the manuscript (Figure 7). Orthogonal methods for apoptosis detection (e.g., Annexin V staining, caspase activation assays, etc.) should be included to validate the findings. Appropriate positive and negative controls are also necessary.

R6. Thank you for this important comment. The manuscript has been revised accordingly. The protein levels of Bax, Bcl-2, and Cleaved Caspase-3 were determined by Western blot analysis (section 3.2 and Figure 3).  Additional apoptosis-related analyses (caspase activation assays) and appropriate controls have been incorporated, and the presentation of the results has been improved for better consistency.

Q7. The experimental design used to assess ER stress appears suboptimal. For the PERK pathway, levels of phosphorylated PERK (p-PERK) or phosphorylated eIF2α (p-eIF2α), normalized to total protein, should be determined; alternatively, CHOP nuclear translocation could be assessed. For the ATF6 pathway, the spliced/activated form of ATF6 (ATF6s) or its translocation should be examined. Additionally, did the authors investigate the IRE1-mediated ER stress pathway?

R7. We appreciate the reviewer’s comment. The assessment of ER stress has now been completed as requested. Specifically, levels of p‑PERK, p‑eIF2α, and the activated form of ATF6 (ATF6s) were measured and normalized to total protein levels. These data have been added to the revised manuscript(Section 3.4 and Figure 6).

**Response to reviewer 1**

Reviewer #1: In the current manuscript, the authors have examined the protective role of protodioscin on cyclophosphamide induced cytotoxicity in renal cells. To improve the quality of the manuscript, the authors need to address the following queries.

Q1. The result section resemble figure legends. It should be rewritten thoroughly.

R1. Thank you for your comment. The necessary revisions have been made throughout the Results section to improve its clarity and presentation.

Q2. Authors have examined the apoptotic genes in early figures while the result from TUNES assay was addressed later in the manuscript. Authors should combine these results as both of them reflect the same conclusion.

R2: We appreciate the reviewer's insightful suggestion. Accordingly, the TUNEL assay results have been relocated to Section 3.3, immediately following the sections describing the apoptosis-related gene and protein expression, so that all apoptosis-related findings are presented together more logically and coherently.

Q3. The study appears to be superficial. Authors need to examine other apoptosis related genes like caspases and their cleaved form through western. The mRNA expression of Bax and Bcl2 is not enough. Protein expression must be examined.

R3. Thank you for this important comment. The manuscript has been revised accordingly. The protein levels of Bax, Bcl-2, and Cleaved Caspase-3 were determined by Western blot analysis (section 3.2 and Figure 3).

Q4. Along with TUNEL, authors should check Annexin V/ PIstaining through FACS to further confirm the apoptosis.

R4: We thank the reviewer for this valuable suggestion. We agree that Annexin V/PI staining followed by flow cytometry is a valuable and widely accepted method for the quantitative assessment of apoptosis. In the present study, however, apoptosis was evaluated using two complementary approaches: the TUNEL assay to detect DNA fragmentation and Western blot analysis of cleaved caspase-3, a well-established marker of apoptotic activation. The consistent findings obtained from these two independent assays provide complementary evidence supporting CTX-induced apoptosis and its attenuation by protodioscin. Although Annexin V/PI flow cytometry would provide additional quantitative confirmation, the experimental phase of this project has been completed, and we are therefore unable to perform additional experiments at this stage. We respectfully hope that the reviewer will agree that the complementary assessment of apoptosis using both TUNEL and cleaved caspase-3 provides sufficient evidence to support our conclusions and kindly request consideration of the revised manuscript in its current form.

Q5. To confirm the observed protective effect of protodioscin on cyclophosphamide induced cytotoxicity in renal cells, authors need to repeat the key data in other kidney cell lines too.

R5. Our study was conducted using the **HEK‑293 cell line**, and we acknowledge that this limits the generalizability of the findings. This limitation has been clearly addressed in the **limitations section** of the Discussion.

**Response to reviewer 2**

Q1. In the methods, the authors mentioned “The data were evaluated using one-way analysis of variance and presented as the mean standard deviation (SD) to compare the experimental groups.”. However, all figures legend showed “Results are presented as mean ± SEM”. There might be contradictions in the preceding and following statements.

R1. It has been corrected.

Q2. Images of TUNEL experiment do not have any scale marks. It should be added

R2. It has been corrected.

Q3. The connecting lines for all the bar charts are somewhat confusing. It is recommended to re-label them.

R3. It has been corrected.

Q4. The subheadings in the results section use the word "kidney cells". To make the research content more clearly and explicitly presented, it is recommended to change to “HEK-293 cells".

R4. It has been corrected

Q5. This study only conducted in vitro experiments and no in vivo experiments. It is suggested that the authors explain the limitations of this study in the discussion and consider adding some relevant literature on in vivo experiments to demonstrate the mechanism of the protodioscin in the body.

R5. Thank you for your constructive and thoughtful evaluation.This point has been addressed and explained in the Limitations, Strengths, and Future Perspectives section of the Discussion

**Best regards,**

**Prof. Cyrus Jalili, Ph.D,**

**Medical Biology Research Center, Health Technology Institute, Kermanshah University of Medical Sciences, Kermanshah, Iran**

**Tel: +98-9188317220, Fax: +98-8334274622**

**Email: cjalili@kums.ac.ir**
