# Supplementary material for: Protodioscin mitigates cyclophosphamide-induced renal toxicity in HEK-293 cells by regulating endoplasmic reticulum stress, antioxidant defense, and apoptotic pathways
Source: PLoS One. 2026 Sep 21;21(9):e0357227. doi: 10.1371/journal.pone.0357227 (PMC13592597; doi:10.1371/journal.pone.0357227)
Supplement: Peer review history

# Peer review: Decision Letter 0

**DOI:** 10.1371/journal.pone.0357227.r001

**Authors:** Irina Lebedeva

11 May 2026

Dear Dr. Jalili,

Thank you for submitting your manuscript to PLOS ONE. After careful consideration, we feel that it has merit but does not fully meet PLOS ONE’s publication criteria as it currently stands. The experiments presented in the manuscript are not sufficient to confirm the authors hypothesis. Therefore, we invite you to submit a revised version of the manuscript that addresses the points raised during the review process.

==============================

**ACADEMIC EDITOR:**

1. The study was conducted using a single cell line, which significantly undermines the generalizability of the conclusions. Given that the manuscript contains exclusively in vitro data, the authors should explicitly discuss the limitations of the study.
2. The Methods section requires additional detail, including the amount of total protein loaded, antibody dilutions, primer sequences, and related experimental parameters. Alternatively, appropriate references to previously published methods should be provided.
3. The Results section is very concise and should be expanded to provide more descriptive interpretation and clearer conclusions from the presented data.
4. Figure 1 – Is the X-axis (CTX concentration) presented on a logarithmic scale?
5. Most experiments lack appropriate positive controls (e.g., apoptosis, oxidative stress, or ER-stress inducers) as well as corresponding inhibitors/negative controls. Their inclusion would strengthen the interpretation of the results.
6. Assessment of Bax and Bcl-2 mRNA levels alone is insufficient; protein expression levels should also be evaluated. In addition, it is unclear why Bax and Bcl-2 measurements are presented in Figure 2 when apoptosis itself is demonstrated only later in the manuscript (Figure 7). Orthogonal methods for apoptosis detection (e.g., Annexin V staining, caspase activation assays, etc.) should be included to validate the findings. Appropriate positive and negative controls are also necessary.
7. The experimental design used to assess ER stress appears suboptimal. For the PERK pathway, levels of phosphorylated PERK (p-PERK) or phosphorylated eIF2α (p-eIF2α), normalized to total protein, should be determined; alternatively, CHOP nuclear translocation could be assessed. For the ATF6 pathway, the spliced/activated form of ATF6 (ATF6s) or its translocation should be examined. Additionally, did the authors investigate the IRE1-mediated ER stress pathway?

==============================

Please submit your revised manuscript by Jun 25 2026 11:59PM. If you will need more time than this to complete your revisions, please reply to this message or contact the journal office at plosone@plos.org. When you're ready to submit your revision, log on to https://www.editorialmanager.com/pone/ and select the 'Submissions Needing Revision' folder to locate your manuscript file.

Please include the following items when submitting your revised manuscript:

- A letter that responds to each point raised by the academic editor and reviewer(s). You should upload this letter as a separate file labeled 'Response to Reviewers'.
- A marked-up copy of your manuscript that highlights changes made to the original version. You should upload this as a separate file labeled 'Revised Manuscript with Track Changes'.
- An unmarked version of your revised paper without tracked changes. You should upload this as a separate file labeled 'Manuscript'.

If you would like to make changes to your financial disclosure, please include your updated statement in your cover letter. Guidelines for resubmitting your figure files are available below the reviewer comments at the end of this letter.

If applicable, we recommend that you deposit your laboratory protocols in protocols.io to enhance the reproducibility of your results. Protocols.io assigns your protocol its own identifier (DOI) so that it can be cited independently in the future. For instructions see: https://journals.plos.org/plosone/s/submission-guidelines#loc-laboratory-protocols. Additionally, PLOS ONE offers an option for publishing peer-reviewed Lab Protocol articles, which describe protocols hosted on protocols.io. Read more information on sharing protocols at https://plos.org/protocols?utm_medium=editorial-email&utm_source=authorletters&utm_campaign=protocols.

As the corresponding author, your ORCID iD is verified in the submission system and will appear in the published article. PLOS supports the use of ORCID, and we encourage all coauthors to register for an ORCID iD and use it as well. Please encourage your coauthors to verify their ORCID iD within the submission system before final acceptance, as unverified ORCID iDs will not appear in the published article. *Only*  the individual author can complete the verification step; PLOS staff *cannot*  verify ORCID iDs on behalf of authors.

We look forward to receiving your revised manuscript.

Kind regards,

Irina V. Lebedeva, Ph.D.

Academic Editor

PLOS One

Journal Requirements:

When submitting your revision, we need you to address these additional requirements.

1. Please ensure that your manuscript meets PLOS ONE's style requirements, including those for file naming. The PLOS ONE style templates can be found at

https://journals.plos.org/plosone/s/file?id=wjVg/PLOSOne_formatting_sample_main_body.pdf and https://journals.plos.org/plosone/s/file?id=ba62/PLOSOne_formatting_sample_title_authors_affiliations.pdf

2. PLOS ONE now requires that authors provide the original uncropped and unadjusted images underlying all blot or gel results reported in a submission’s figures or Supporting Information files. This policy and the journal’s other requirements for blot/gel reporting and figure preparation are described in detail at https://journals.plos.org/plosone/s/figures#loc-blot-and-gel-reporting-requirements and https://journals.plos.org/plosone/s/figures#loc-preparing-figures-from-image-files. When you submit your revised manuscript, please ensure that your figures adhere fully to these guidelines and provide the original underlying images for all blot or gel data reported in your submission. See the following link for instructions on providing the original image data: https://journals.plos.org/plosone/s/figures#loc-original-images-for-blots-and-gels.

In your cover letter, please note whether your blot/gel image data are in Supporting Information or posted at a public data repository, provide the repository URL if relevant, and provide specific details as to which raw blot/gel images, if any, are not available. Email us at plosone@plos.org if you have any questions.

3. PLOS requires an ORCID iD for the corresponding author in Editorial Manager on papers submitted after December 6th, 2016. Please ensure that you have an ORCID iD and that it is validated in Editorial Manager. To do this, go to ‘Update my Information’ (in the upper left-hand corner of the main menu), and click on the Fetch/Validate link next to the ORCID field. This will take you to the ORCID site and allow you to create a new iD or authenticate a pre-existing iD in Editorial Manager.

4. Your ethics statement should only appear in the Methods section of your manuscript. If your ethics statement is written in any section besides the Methods, please move it to the Methods section and delete it from any other section. Please ensure that your ethics statement is included in your manuscript, as the ethics statement entered into the online submission form will not be published alongside your manuscript.

5. Please provide a complete Data Availability Statement in the submission form, ensuring you include all necessary access information or a reason for why you are unable to make your data freely accessible. If your research concerns only data provided within your submission, please write "All data are in the manuscript and/or supporting information files" as your Data Availability Statement.

6. When completing the data availability statement of the submission form, you indicated that you will make your data available on acceptance. We strongly recommend all authors decide on a data sharing plan before acceptance, as the process can be lengthy and hold up publication timelines. Please note that, though access restrictions are acceptable now, your entire data will need to be made freely accessible if your manuscript is accepted for publication. This policy applies to all data except where public deposition would breach compliance with the protocol approved by your research ethics board. If you are unable to adhere to our open data policy, please kindly revise your statement to explain your reasoning and we will seek the editor's input on an exemption. Please be assured that, once you have provided your new statement, the assessment of your exemption will not hold up the peer review process.

7. Please update your submission to use the PLOS LaTeX template. The template and more information on our requirements for LaTeX submissions can be found at http://journals.plos.org/plosone/s/latex.

8. If the reviewer comments include a recommendation to cite specific previously published works, please review and evaluate these publications to determine whether they are relevant and should be cited. There is no requirement to cite these works unless the editor has indicated otherwise.

[Note: HTML markup is below. Please do not edit.]

Reviewers' comments:

Reviewer's Responses to Questions

**Comments to the Author**

1. Is the manuscript technically sound, and do the data support the conclusions?

Reviewer #1: Partly

Reviewer #2: Yes

**********

2. Has the statistical analysis been performed appropriately and rigorously?

Reviewer #1: Yes

Reviewer #2: Yes

**********

3. Have the authors made all data underlying the findings in their manuscript fully available?

The PLOS Data policy

Reviewer #1: Yes

Reviewer #2: Yes

**********

4. Is the manuscript presented in an intelligible fashion and written in standard English?

Reviewer #1: Yes

Reviewer #2: Yes

**********

Reviewer #1: In the current manuscript, the authors have examined the protective role of protodioscin on cyclophosphamide induced cytotoxicity in renal cells. To improve the quality of the manuscript, the authors need to address the following queries.

1. The result section resemble figure legends. It should be rewritten thoroughly.

2. Authors have examined the apoptotic genes in early figures while the result from TUNES assay was addressed later in the manuscript. Authors should combine these results as both of them reflect the same conclusion.

3. The study appears to be superficial. Authors need to examine other apoptosis related genes like caspases and their cleaved form through western. The mRNA expression of Bax and Bcl2 is not enough. Protein expression must be examined.

4. Along with TUNEL, authors should check Annexin V/ PI staining through FACS to further confirm the apoptosis.

5. To confirm the observed protective effect of protodioscin on cyclophosphamide induced cytotoxicity in renal cells, authors need to repeat the key data in other kidney cell lines too.

Reviewer #2: In this manuscript, the authors investigated the protective potential of protodioscin against CTX-induced damage in the HEK-293 cell line and generated some interesting data, however, there are some flaws in the text and there are major revisions should be done before the manuscript is accepted for publication.

1.In the methods, the authors mentioned “The data were evaluated using one-way analysis of variance and presented as the mean standard deviation (SD) to compare the experimental groups.”. However, all figures legend showed “Results are presented as mean ± SEM”. There might be contradictions in the preceding and following statements.

2.Images of TUNEL experiment do not have any scale marks. It should be added

3.The connecting lines for all the bar charts are somewhat confusing. It is recommended to re-label them.

4.The subheadings in the results section use the word "kidney cells". To make the research content more clearly and explicitly presented, it is recommended to change to " HEK-293 cells".

5.This study only conducted in vitro experiments and no in vivo experiments. It is suggested that the authors explain the limitations of this study in the discussion and consider adding some relevant literature on in vivo experiments to demonstrate the mechanism of the protodioscin in the body.

**********

what does this mean?). If published, this will include your full peer review and any attached files.

If you choose “no”, your identity will remain anonymous but your review may still be made public.

**Do you want your identity to be public for this peer review?**  For information about this choice, including consent withdrawal, please see our Privacy Policy

Reviewer #1: **Yes:** Dipankar Ash

Reviewer #2: No

**********

[NOTE: If reviewer comments were submitted as an attachment file, they will be attached to this email and accessible via the submission site. Please log into your account, locate the manuscript record, and check for the action link "View Attachments". If this link does not appear, there are no attachment files.]

To ensure your figures meet our technical requirements, please review our figure guidelines: https://journals.plos.org/plosone/s/figures

You may also use PLOS’s free figure tool, NAAS, to help you prepare publication quality figures: https://journals.plos.org/plosone/s/figures#loc-tools-for-figure-preparation.

NAAS will assess whether your figures meet our technical requirements by comparing each figure against our figure specifications.

# Author comment: Author response to Decision Letter 1

**DOI:** 10.1371/journal.pone.0357227.r002

9 Jul 2026

My co-authors and I sincerely appreciate the reviewers’ encouraging, critical, and constructive comments on our manuscript entitled “Protodioscin Mitigates Cyclophosphamide-Induced Renal Toxicity in HEK-293 Cells by regulating Antioxidant and Apoptotic Pathways”. In response to their recommendations, and considering the limitations of time and available experimental facilities, we performed additional Western blot analyses for the apoptosis-related proteins BAX, BCL-2, and cleaved caspase-3, as well as the ER stress signaling proteins cleaved ATF6, phosphorylated PERK (p-PERK), and phosphorylated eIF2α (p-eIF2α). These new data have been incorporated into the revised manuscript. In addition, H₂O₂ was included as a positive control in the apoptosis and TUNEL assays, and a graphical abstract has been added to the revised manuscript. We are grateful for the insightful feedback and valuable suggestions that have helped us improve the quality and clarity of our paper.

Attachment Submitted filename: letter (3).docx

# Peer review: Decision Letter 1

**DOI:** 10.1371/journal.pone.0357227.r003

**Authors:** Irina Lebedeva

14 Aug 2026

Protodioscin Mitigates Cyclophosphamide-Induced Renal Toxicity in HEK-293 Cells by Regulating Endoplasmic Reticulum Stress, Antioxidant Defense, and Apoptotic Pathways

PONE-D-26-08024R1

Dear Dr. Jalili,

We’re pleased to inform you that your manuscript has been judged scientifically suitable for publication and will be formally accepted for publication once it meets all outstanding technical requirements.

Within one week, you’ll receive an e-mail detailing the required amendments. When these have been addressed, you’ll receive a formal acceptance letter and your manuscript will be scheduled for publication.

An invoice will be generated when your article is formally accepted. Please note, if your institution has a publishing partnership with PLOS and your article meets the relevant criteria, all or part of your publication costs will be covered. Please make sure your user information is up-to-date by logging into Editorial Manager at Editorial Manager® and clicking the ‘Update My Information' link at the top of the page. For questions related to billing, please contact billing support.

If your institution or institutions have a press office, please notify them about your upcoming paper to help maximize its impact. If they’ll be preparing press materials, please inform our press team as soon as possible -- no later than 48 hours after receiving the formal acceptance. Your manuscript will remain under strict press embargo until 2 pm Eastern Time on the date of publication. For more information, please contact onepress@plos.org.

Kind regards,

Irina V. Lebedeva, Ph.D.

Academic Editor

PLOS One

Additional Editor Comments (optional):

Reviewers' comments:

Reviewer's Responses to Questions

**Comments to the Author**

Reviewer #1: All comments have been addressed

Reviewer #2: All comments have been addressed

**********

2. Is the manuscript technically sound, and do the data support the conclusions?

Reviewer #1: (No Response)

Reviewer #2: Yes

**********

3. Has the statistical analysis been performed appropriately and rigorously?

Reviewer #1: (No Response)

Reviewer #2: Yes

**********

4. Have the authors made all data underlying the findings in their manuscript fully available?

The PLOS Data policy

Reviewer #1: (No Response)

Reviewer #2: Yes

**********

5. Is the manuscript presented in an intelligible fashion and written in standard English?

Reviewer #1: (No Response)

Reviewer #2: Yes

**********

Reviewer #1: (No Response)

Reviewer #2: This manuscript investigated the potential of protodioscin against CTX-induced damage in the HEK-293 cell line and generated some interesting data.These data suggest that Prot exerts a protective role against CTX-induced renal injury in HEK-293 cells by modulating apoptotic pathways, attenuating endoplasmic reticulum stress, and improving antioxidant indices and may provide some new ideas for the prevention and treatment of kidney damage induced by CTX.

**********

what does this mean?). If published, this will include your full peer review and any attached files.

If you choose “no”, your identity will remain anonymous but your review may still be made public.

**Do you want your identity to be public for this peer review?**  For information about this choice, including consent withdrawal, please see our Privacy Policy

Reviewer #1: **Yes:** Dipankar Ash

Reviewer #2: No

**********

# Editor report: Acceptance letter

**DOI:** 10.1371/journal.pone.0357227.r004

**Authors:** Irina Lebedeva

PONE-D-26-08024R1

PLOS One

Dear Dr. Jalili,

I'm pleased to inform you that your manuscript has been deemed suitable for publication in PLOS One. Congratulations! Your manuscript is now being handed over to our production team.

At this stage, our production department will prepare your paper for publication. This includes ensuring the following:

* All references, tables, and figures are properly cited

* All relevant supporting information is included in the manuscript submission,

* There are no issues that prevent the paper from being properly typeset

You will receive further instructions from the production team, including instructions on how to review your proof when it is ready. Please keep in mind that we are working through a large volume of accepted articles, so please give us a few days to review your paper and let you know the next and final steps.

Lastly, if your institution or institutions have a press office, please let them know about your upcoming paper now to help maximize its impact. If they'll be preparing press materials, please inform our press team within the next 48 hours. Your manuscript will remain under strict press embargo until 2 pm Eastern Time on the date of publication. For more information, please contact onepress@plos.org.

You will receive an invoice from PLOS for your publication fee after your manuscript has reached the completed accept phase. If you receive an email requesting payment before acceptance or for any other service, this may be a phishing scheme. Learn how to identify phishing emails and protect your accounts at https://explore.plos.org/phishing.

If we can help with anything else, please email us at customercare@plos.org.

Thank you for submitting your work to PLOS One and supporting open access.

Kind regards,

PLOS One Editorial Office Staff

on behalf of

Dr. Irina V. Lebedeva

Academic Editor

PLOS One
